# Une cataracte inhabituelle: régressive à noyau pétaloïde

**DOI:** 10.11604/pamj.2018.31.220.16017

**Published:** 2018-12-04

**Authors:** Ahmed Bennis, Idriss Andaloussi Benatiya

**Affiliations:** 1 Centre Hospitalier Hassan II, Faculté de Médecine et de Pharmacie de Fès, Maroc; 2 Centre Hospitalier Hassan II, Service d’Ophtalmologie, Hôpital Omar Drissi, Fès, Maroc

**Keywords:** Cataracte, traumatisme contusif, pétaloïde, Cataract, contusive trauma, petaloid

## Abstract

We report the case of a 38-year old patient with no previous medical-surgical history, victim of traumatic contusion injury in the right eye resulting from stone blow which occurred 20 years before and experiencing progressive decrease in visual acuity over the last 10 years. Ophthalmologic examination of the right eye showed visual acuity of counting fingers and ocular tone 11 mmHg. Examination of the anterior segment after dilation of the pupil showed regressive cataract with petaloid-shaped crystalline core. The petals had dense edges and sutures. Given the inaccessibility of the ocular fundus and that the examination of the left eye objectified no abnormalities, ocular ultrasound was performed.

## Image en médecine

Nous rapportons le cas d'une patiente de 38 ans, sans antécédents médico-chirurgicaux, victime d'un traumatisme contusif de l'œil droit par coup de pierre il y a 20 ans, avec baisse de l'acuité visuelle progressive depuis 10 ans. L'examen ophtalmologique note au niveau de l'œil droit une acuité visuelle à mouvement des doigts et un tonus oculaire à 11mmHg. L'examen du segment antérieur après dilatation objective une cataracte régressive, dont le noyau présumé est de forme pétaloïde, les bords et les sutures des pétales sont denses. Une échographie oculaire est réalisée vu l'inaccessibilité du fond d'œil, ainsi que l'examen de l'œil gauche n'objective aucune anomalie.

**Figure 1:**
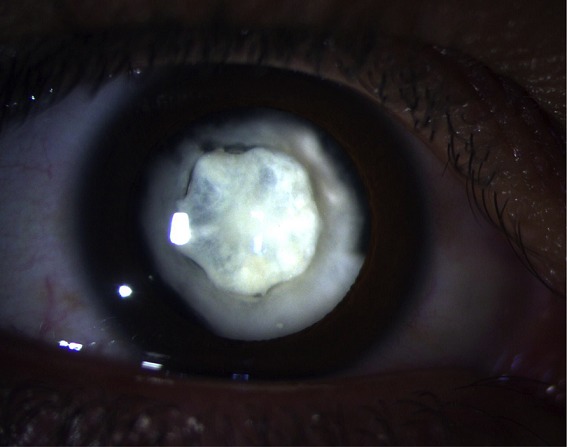
image bio microscopique de l'oeil droit à pupille dilatée objectivant une cataracte régressive dont le moignon résiduelle est de forme pétaloïde

